# Factors associated with informal caregiving and its effects on health, work, and social activities of adult informal caregivers in Malaysia: findings from the National Health and Morbidity Survey 2019

**DOI:** 10.1186/s12889-021-11022-1

**Published:** 2021-06-01

**Authors:** Yuke-Lin Kong, Jailani Anis-Syakira, Suhana Jawahir, Yeung R’ong Tan, Noor Hasidah Ab Rahman, Ee Hong Tan

**Affiliations:** Institute for Health Systems Research, National Institutes of Health, Ministry of Health Malaysia, Block B2, NIH Complex, No. 1, Jalan Setia Murni U13/52, Section U13 Setia Alam, 40170 Shah Alam, Selangor Malaysia

**Keywords:** Caregivers, Informal care, Effects, Health, Work, Malaysia

## Abstract

**Background:**

The increase in the elderly population, chronic and degenerative diseases, as well as accidents at work and on the road in Malaysia would result in an increased demand for informal care. This paper aimed to determine the associated factors of informal caregiving and its effects on health, work and social activities of adult informal caregivers in Malaysia.

**Methods:**

The data from the 2019 National Health and Morbidity Survey (NHMS), a nationwide cross-sectional survey with a two-stage stratified random sampling design, was used in this research. The study included respondents who were 18 years and older (*n* = 11,674). Data were obtained via face-to-face interviews using validated questionnaires. Descriptive and complex sample logistic regression analyses were employed as appropriate.

**Results:**

5.7% of the adult population were informal caregivers. Provision of informal care were significantly associated with the female sex (OR = 1.52, 95% CI [1.21, 1.92]), those aged 36–59 years (OR = 1.61, 95% CI [1.15, 2.25]), and those who reported illness in the past 2 weeks (OR = 1.79, 95% CI [1.38, 2.33]). The risk of having their health affected were associated with female caregivers (OR = 3.63, 95% CI [1.73, 7.61]), those who received training (OR = 2.10, 95% CI [1.10, 4.00]) and those who provided care for 2 years or more (OR = 1.91, 95% CI [1.08, 3.37]). The factors associated with the effects on work were ethnicity, received training and had no assistance to provide the care. In terms of effect on social activities, female caregivers (OR = 1.96, 95% CI [1.04, 3.69]) and caregivers who received training were more likely (OR = 2.19, 95% CI [1.22, 3.93]) to have their social activities affected.

**Conclusion:**

Our study revealed that sex, age, and self-reported illness were factors associated with being an informal caregiver in Malaysia. Informal caregivers faced effects on their health, work, and social activities which may be detrimental to their well-being. This understanding is crucial for planning support for caregivers.

## Background

Informal care is the provision of unpaid care or support to others who need help or who are unable to care for themselves due to long term health conditions and disability [1]. The care may involve assistance with walking, feeding, dressing, toileting, bathing, accessing healthcare services, managing medications and also housekeeping. Many would have contributed informal care at one point or another in their lives. Informal caregivers could be family members, relatives, friends or even neighbours, except care provided by professionals or through organised voluntary services [2]. Informal care has significant preventative properties in terms of avoiding or delaying institutionalisation [3] and can be seen as a practical measure to contain the costs of health services while at the same time support the widespread preferences among older people to be cared for at their own home and their familiar environment [4, 5].

Malaysia is expected to be an ageing nation by 2030 [6]. The ageing population along with the rise in incidence of chronic and degenerative diseases such as diabetes, hypertension, cardiovascular diseases, and stroke, as well as elevated cases of accidents at work and on the road in Malaysia [7] points towards an increased need for informal care provision [8]. However, the overall prevalence of informal caregivers in Malaysia (5.3% of the population) was low [9] compared to other countries (USA - 28.5% of adults aged 18 years and over, Singapore - 8.1% of adults aged 18–69 years) [10, 11]. Massive urban migration among younger adults for better job opportunities, financial constraints due to inflation and increasing cost of living in the cities, lack of preparation to shoulder caregiving responsibilities due to late diagnosis of disease such as cancer, and cultural resentment over caregiving responsibilities among family members have been reported as barriers of caregiving [12]. In addition, the increasing age at marriage, longer lifespan, participation of women in the labour force, smaller household size, and the growing preference for the nuclear family concept led to difficulty in provision of informal care [13].

Filial obligation is a cultural norm in Asia where family members are socially assigned, morally obliged and intrinsically assumed to care for the unwell family member [12, 14, 15]. In Malaysia, many of the older generation live together and were provided care by their children especially after their spouse has deceased [14]. This may be supplemented or sometimes replaced by a live-in foreign paid domestic helper from a neighbouring Southeast Asian country for those who can afford it [16, 17]. The Malaysian government introduced the Community Care Policy in 1990 to assist communities who need care and support due to illness or disability as well as their caregivers [18] but minimal provision of services led to inadequate resources and caregivers support [7].

The care process is dynamic in nature and involves the interaction of multiple actors; the care recipient, caregivers, and professionals. Theoretical models have attempted to conceptualise the factors associated with provision of informal care but many focus on caregiving outcomes [19] rather than socio-psychological processes and societal context. The informal care model by Broese van Groenou and De Boer however, focus on the individual caregiver and consists of three central propositions; the care recipient’s need for care, individual dispositional factors to predict intention to provide care, and external conditions that facilitates or restricts the provision of care [20]. According to this behavioural theory, informal care provision is an interface between individual, relational, and contextual factors of the care recipient and caregiver [21]. The need for care includes physical and mental health of the care recipient while dispositional factors include attitude and affection, norms of solidarity and reciprocity, as well as perceived barriers of distance, time, money and competence of the caregivers. External conditions that facilitate or restricts provision of care could be contextual factors such as presence of other helpers or assistance, family and social network factors, and availability of community care services [21].

Numerous studies have reported the factors which influences informal care in developed and developing countries. The need for care such as care recipient’s health status [22], dispositional factors such as age, gender, ethnicity, education, region, annual income, marital or partnership status, employment, civil status, religion, relationship to patients, and contextual factors such as presence of community care services and family or social network support have been found to contribute to the likelihood of informal caregiving [23–25]. The care recipient’s health status triggers the need for care which leads to the utilisation of care [22], while individual dispositional factors determines the intention or ability to provide care. Furthermore, caregiving requires commitment and sacrifices to be made which may inflict changes in the caregiver’s physical, mental, financial, and social well-being, both positively and negatively [17, 24–31]. For instance, the ability to provide care decreases as age increases due to a decline in strength and mobility in carrying tasks [32] while low-income caregivers subjectively felt more overburdened with their caregiving responsibilities compared to higher income caregivers due to differences in literacy level and financial difficulties [33]. Contextual factors such as presence of other family members or social network to share caregiving responsibilities facilitates provision of informal care while absence of a support system such as community services restricts the provision of care.

There is a dearth of information regarding factors associated with informal caregiving and its effect on informal caregivers in Malaysia. One study identified the consequences of caregiving responsibilities to the financial, social, physical and mental well-being of informal caregivers but included only respondents from two states in Malaysia [17]. Hence, the findings could not be generalised across the population in Malaysia. As the need for informal care provision rises, understanding the interaction of the various factors which influences informal care provision becomes crucial as it could help formulate effective and efficient policies which support informal care provision in Malaysia. To the best of our knowledge, a study pertaining to informal care provision that could be generalised nationally is currently unavailable in Malaysia. To fill this knowledge gap, our study aims to determine the factors associated with informal care provision and identify how these factors affect health, work and social activities of adult informal caregivers in Malaysia using the informal care model.

## Methods

### Study design and sampling

This study analysed data of adults aged 18 years and above from the National Health and Morbidity Survey (NHMS) 2019, a cross-sectional nationwide household survey that targeted the non-institutionalised Malaysia population. Selection of samples was conducted based on the sampling frame provided by the Department of Statistics of Malaysia (DOSM) using the National Population and Housing Census 2010. The geographical areas of Malaysia were divided into Enumeration Blocks (EBs) based on the frame. In Malaysia, there were around 75,000 EBs, with each EB containing 80 to 120 Living Quarters (LQs) and a population of 500 to 600 people. In order to ensure national representativeness, the two-stage stratified sampling was adopted in the survey. The two strata were the primary stratum, which was made up of states and federal territories in Malaysia, and the second stratum, which was made up of urban and rural strata formed within the states. All states and federal territories were included in the survey, and within each state, selected number of EBs from urban and rural areas were randomly selected. The number of samples allocated for each state, urban and rural was done proportionally to the population size. The Primary Sampling Unit (PSU) is Enumeration Block (EB). A total of 463 EBs were selected from the total EBs in Malaysia, where 350 and 113 EBs were selected from urban and rural areas, respectively. The Secondary Sampling Unit (SSU) is LQs within the selected EBs. Fourteen LQs were randomly selected from each selected EBs. All households within the selected LQs were included in the study. All eligible members in the households were also included in the study. The overall response rate for this community-based survey is therefore 83.4%. A detailed description of the methods and sampling design of the survey is described in the NHMS 2019 technical report [9]. A total of 11,674 eligible adults aged 18 years and above were invited to participate in this survey.

### Study instrument

The healthcare demand questionnaire of NHMS 2019 comprised of 10 topics: 1) household, 2) sociodemographic and socioeconomic, 3) payer for healthcare, 4) general health & illness, 5) utilisation of community pharmacy, 6) utilisation of outpatient healthcare, 7) utilisation of inpatient healthcare, 8) utilisation of oral healthcare, 9) home-visit, and 10) informal care [34].

### Data collection

Data were collected from July to October 2019 by trained interviewers using a pre-tested and validated questionnaire [9, 34]. The questionnaire was programmed into an application and uploaded onto tablets used as mobile data collection devices. The tablets were used to collect data, store and back up data in the Secure Digital cards, and upload data to the central system. Prior to data collection, a training course was conducted for the teams, which consisted of field supervisors, team leaders, nurses, and interviewers. Houses that were empty or closed during the initial visit were revisited up to three times to ensure the minimum required sample size is achieved. Information sheets were given to all eligible adults. Informed consent was obtained prior to the interview.

### Explanatory variables

#### Dependent variables

Provision of informal care was measured using a question which query about provision of healthcare, personal and other types of care to household members and/or non-household members with long-term health conditions (such as stroke, diabetes, kidney disease, heart conditions, mental illness, dementia and others), elderly or those unable to care for themselves in the last 12 months prior to the interview. Respondents were considered as informal caregivers if they have provided informal care for at least 3 months without involvement of wage or salary, community service and voluntary activity. In this paper, ‘caregivers’ will be used to refer to informal caregivers. The other key outcome variables were the effect of caring roles on health, work, and social activities of caregivers. The effects were examined as a dichotomous variable (yes/no) if their role in providing care affected: 1) health (physical and/or mental health), 2) daily, work or school activities, and 3) social activities and others. In this paper, ‘work’ will be used to refer to daily, work or school activities.

#### Independent variables

##### Care recipient’s need for care

Care recipient’s need for care was described by type of care provided: 1) healthcare; and 2) personal care and others.

##### Individual dispositional factors

Individual dispositional factors were described by sex, age, marital status, ethnicity, education level, employment status, income, residency, received training, duration of care, intensity of care (in a week), self-reported illness, perceived health status and presence of diabetes mellitus (DM), hypertension (HPT), or hypercholesterolemia. Age of caregivers in years was grouped into “18–35”, “36–59” and “60 and above” based on age distribution pattern. Ethnicity was grouped into “Malay” for Malay ethnicity and “non-Malay” for ethnicity other than Malay. Malays are the main ethnic group in Malaysia which made up of 69.6% of the population [35]. Income was calculated based on monthly household income and analysed as quintile. The quintile was then grouped into three income groups; “low” (first quintile and second quintile), “middle” (third quintile and fourth quintile), and “high” (fifth quintile). This grouping was made based on the household income categories in Malaysia, namely the Top 20% (T20), Middle 40% (M40), and Bottom 40% (B40). To assess if the caregivers received training for their caregiving roles, the respondents were asked if they were trained to provide care to the care recipient by a healthcare practitioner, other than healthcare practitioner, or no training was received. Duration of care was assessed by the question “How long have you been providing care to the care recipient?”. The number of years was then grouped into two categories; less than 2 years and 2 years and more [26]. For intensity of care (in a week), the respondents were asked to answer the question “In total, how many hours per week did you normally spend providing care to the care recipient? (Estimation)” [8]. For the analysis, respondents were grouped into “19 hours and less” and “20 hours and above” [23]. For self-reported illness, the respondents were asked if they experienced any of the following health problems such as fever, sore throat, difficulty in swallowing, running nose or blocked nose, cough, and others, within 2 weeks prior to the interview. Respondents were asked to rate their health status in general, using a five-point scale (excellent, good, fair, poor, very poor) for the variable perceived health status. The responses were then grouped into two categories; “very poor to poor” (very poor or poor), and “fair to excellent” (fair or good or excellent). For the presence of DM, HPT, and/or hypercholesterolemia, the respondents were required to respond (yes/no) if they have ever been told by doctor(s) or Assistant Medical Officer(s) that they have DM, HPT, or hypercholesterolemia.

##### Contextual factors

Contextual factors were represented by the variables ‘living in the same household’ and ‘had assistance’. To assess if the caregivers had assistance for their caregiving tasks, the respondents were required to answer (yes/no) if anyone else such as other family members, domestic helper/maid, nurse/other nursing professional, day-care/other institution, or others (e.g. neighbours) provided the care to the care recipient. For all the independent and dependent variables measured, respondents who answered don’t know or refused to answer, the responses were coded as “missing”.

### Statistical analysis

Analysis of data was done using STATA version 14 (Stata Corp, College Station, Texas, USA), and sample weights and study design were taken into consideration using a complex sampling design in all data analyses using the survey (svy) command. The weight used for estimation was based on the products of the inverse of the probability of sampling, non-response adjustment factor, and a post-stratification adjustment by age, gender, and ethnicity. Complex sample descriptive statistics were used to illustrate the characteristics of the caregivers and non-caregivers. The factors associated with provision of informal care and associations between demographic, socio-economic, health-related and caregiving-related characteristics of the informal caregivers with effect on health, work, and social activities were assessed using logistic regression analysis. Variables with *p*-value < 0.25 [36] in univariate analysis were included in the multivariable regression analysis. Multivariable logistic regression model was fitted to determine association between categorical dependent variables of informal care provision as well as effect on their health, work, and social activities with the informal caregiver’s demographic, socio-economic, health-related and caregiving-related characteristics Adjusted OR with 95% CI were determined and *p*-value < 0.05 was considered statistically significant. Collinearity of variables was assessed by applying coldiag2 command in Stata to generate a condition index. The threshold for condition index was set at 30, where condition index of 30 and more indicates multicollinearity problems and require further assessment [37]. Receiver operational characteristic curves and areas under the curve (AUC) were used to evaluate the model’s goodness of fit. An AUC of 0.9–1.0 was considered excellent, 0.8–0.9 very good, 0.7–0.8 good, 0.6–0.7 sufficient, 0.5–0.6 bad, and less than 0.5 not useful [38].

## Results

### Characteristics of informal caregivers and non-caregivers

Table 1 shows the characteristics of the respondents. A total of 11,674 respondents aged 18 years and over were included in this analysis. Of those, 699 (5.7%) were informal caregivers and 10,975 (94.3%) were non-caregivers. Caregivers were mainly women (61.7%), aged 36–59 (48.8%), married (67.6%), Malay (56.5%), and had secondary level education (52.7%). Caregivers provided care mainly to household members (85.0%), majority of them received no training (76.6%), had assistance (67.6%), provided care for 2 years and more (63.8%) and provided care for 19 h and less in a week (57.1%).

**Table 1:** Characteristics of informal caregivers and non-caregivers (*N* = 11,674)

Characteristic	Overall	Caregivers	Non-caregivers	***p***-value
	n (weighted %)	n (weighted %)	n (weighted %)	
***Care recipient’s need for care***				
**Type of care provided**				
**Healthcare** ^***a***^				
Yes	592 (83.6)	592 (83.6)	*n/a*	*–*
No	84 (11.8)	84 (11.8)	*n/a*	*–*
**Personal care and others** ^***a***^				
No	550 (19.2)	550 (19.2)	*n/a*	*–*
Yes	125 (76.1)	125 (76.1)	*n/a*	*–*
***Individual dispositional factors***				
**Sex**				
Male	5517 (49.9)	275 (38.3)	5242 (50.6)	< 0.001
Female	6157 (50.1)	424 (61.7)	5733 (49.4)	
**Age group (years)**				
18–35	3729 (44.4)	149 (32.7)	3580 (45.1)	< 0.001
36–59	5441 (40.8)	376 (48.8)	5065 (40.3)	
60 and above	2504 (14.8)	174 (18.5)	2330 (14.5)	
**Marital status**^**a**^				
Not married	3738 (37.0)	201 (32.4)	3537 (37.3)	0.055
Married	7927 (62.9)	498 (67.6)	7429 (62.6)	
**Ethnicity**				
Malay	7642 (51.3)	469 (56.5)	7173 (51.0)	0.349
Non-Malay	4032 (48.7)	230 (43.5)	3802 (49.0)	
**Education level**^**a**^				
No formal education	679 (5.4)	38 (5.9)	641 (5.4)	0.044
Primary	2540 (19.7)	162 (19.0)	2378 (19.7)	
Secondary	5554 (48.7)	357 (52.7)	5197 (48.5)	
Tertiary	2862 (25.8)	142 (22.4)	2720 (26.0)	
**Employment status**^**a**^				
No	4884 (37.8)	338 (45.5)	4546 (37.4)	< 0.001
Yes	6781 (62.1)	361 (54.5)	6420 (62.6)	
**Household income group**^**a**^				
Low	4791 (40.3)	343 (46.8)	4448 (39.9)	< 0.001
Middle	4551 (39.6)	261 (38.1)	4290 (39.7)	
High	2247 (19.4)	93 (15.0)	2154 (19.7)	
**Residency**				
Urban	7015 (75.6)	424 (73.5)	6591 (75.7)	0.752
Rural	4769 (24.3)	275 (26.5)	4384 (24.3)	
**Received training**^**a**^				
No	531 (76.6)	531 (76.6)	*n/a*	–
Yes	142 (18.4)	142 (18.4)	*n/a*	–
**Duration of care**				
Less than 2 years	237 (36.2)	237 (36.2)	*n/a*	–
2 years and more	462 (63.8)	462 (63.8)	*n/a*	–
**Intensity of care (in a week)**^**a**^				
19 h and less	410 (57.1)	410 (57.1)	*n/a*	–
20 h and above	236 (35.1)	236 (35.1)	*n/a*	–
**Self-reported illness**^**a**^				
No	8900 (79.0)	468 (67.3)	8432 (79.7)	< 0.001
Yes	2747 (20.8)	230 (32.6)	2517 (20.1)	
**Perceived health status**^**a**^				
Very poor to poor	2842 (20.7)	199 (26.4)	2643 (20.3)	0.007
Fair to excellent	8751 (78.5)	493 (72.5)	8258 (78.9)	
**Presence of DM, HPT or HPC**^**a**^				
No	8142 (20.7)	449 (68.9)	7693 (76.4)	0.001
Yes	3311 (78.5)	237 (28.6)	3074 (21.5)	
***Contextual factors***				
**Living in the same household**				
No	93 (15.0)	93 (15.0)	*n/a*	–
Yes	606 (85.0)	606 (85.0)	*n/a*	–
**Had assistance**				
No	217 (32.4)	217 (32.4)	*n/a*	–
Yes	482 (67.6)	482 (67.6)	*n/a*	–

*n/a* not applicable, *DM* Diabetes Mellitus, *HPT* Hypertension, *HPC* Hypercholesterolemia; ^*a*^ Consists of missing values (< 5%)

### Factors associated with provision of informal care

The associated factors of informal caregiving were sex, age group and self-reported illness (Table 2). Females were 1.52 (95% CI: 1.21–1.92) times more likely to be caregivers as compared to males. Those in the age group of 36–59 years were 1.61 (95% CI: 1.15–2.25) times more likely to be caregivers as compared to the younger age group of 18–35 years. Those reported experienced illness in the past 2 weeks prior to the interview were 1.79 (95% CI: 1.38–2.33) times more likely to provide informal care as compared to those who did not.

**Table 2:** Logistic regression analysis of factors associated with informal caregiving

Factor	Crude OR (95% CI)	Adjusted OR (95% CI)
**Sex**		
Male	*Ref*	*Ref*
Female	1.65 (1.34–2.04)	1.52 (1.21–1.92)***
**Age group (years)**		
18–35	*Ref*	*Ref*
36–59	1.67 (1.25–2.23)	1.61 (1.15–2.25)**
60 and above	1.76 (1.23–2.52)	1.48 (0.97–2.24)
**Marital status**		
Not married	*Ref*	*Ref*
Married	1.25 (0.94–1.65)	1.05 (0.76–1.45)
**Ethnicity**		
Malay	*Ref*	*Ref*
Non-Malay	0.80 (0.61–1.05)	0.83 (0.63–1.09)
**Education level**^**a**^		
No formal education	1.28 (0.71–2.30)	
Primary	1.11 (0.76–1.63)	
Secondary	1.26 (0.94–1.69)	
Tertiary	*Ref*	–
**Employment status**		
No	1.34 (1.11–1.76)	1.11 (0.84–1.48)
Yes	*Ref*	*Ref*
**Household income group**		
Low	1.36 (0.98–1.88)	1.23 (0.86–1.74)
Middle	1.10 (0.77–1.59)	1.13 (0.78–1.63)
High	*Ref*	*Ref*
**Residency**^**a**^		
Urban	0.89 (0.70–1.13)	
Rural	*Ref*	–
**Self-reported illness**		
No	*Ref*	*Ref*
Yes	1.91 (1.49–2.47)	1.79 (1.38–2.33)***
**Perceived health status**		
Very poor to poor	*Ref*	*Ref*
Fair to excellent	1.41 (1.09–1.83)	1.00 (0.72–1.39)
**Presence of DM, HPT or HPC**		
No	*Ref*	*Ref*
Yes	1.48 (1.14–1.90)	1.07 (0.80–1.42)

*CI* Confidence Interval, *OR*, Odds Ratio, *Ref* Reference category, *DM* Diabetes Mellitus, *HPT* Hypertension, *HPC* Hypercholesterolemia; ^a^ *p*-value > 0.25 for univariate analysis; * *p*-value < 0.05; ** *p*-value < 0.01; *** *p*-value < 0.001; Area under ROC curve for model provision of informal care = 0.6364

### Factors associated with the effect on health, work, and social activities among informal caregivers

The univariate and multivariable analysis for factors associated with the effects on health of the informal caregivers are shown in Table 3. The factors associated with the effects on health were sex, received training and duration of care provided. Female caregivers were 3.63 (95% CI: 1.73–7.61) times more likely to have their health affected as compared to male caregivers. Caregivers who received training and provided care for 2 years and more were 2.10 (95% CI: 1.10–4.00) and 1.91 (95% CI: 1.08–3.37) times more likely to have their health affected, as compared to caregivers who did not receive training and provided care for less than 2 years, respectively.

**Table 3:** Logistic regression analysis of effect on health, work and social activities among informal caregivers

Factor	Effect on health		Effect on work		Effect on social activities	
	Crude OR (95% CI)	Adjusted OR (95% CI)	Crude OR (95% CI)	Adjusted OR (95% CI)	Crude OR (95% CI)	Adjusted OR (95% CI)
***Care recipient’s need for care***						
**Type of care provided**						
**Healthcare**^**a,b,c**^						
Yes	1.03 (0.41, 2.65)		1.41 (0.62, 3.18)		1.24 (0.53, 2.90)	
No	*Ref*		*Ref*		*Ref*	
**Personal care and others**^**b,c**^						
Yes	2.13 (0.63, 7.32)	2.25 (0.62,8.20)	1.55 (0.65, 3.69)		1.46 (0.62, 3.41)	
No	*Ref*	*Ref*	*Ref*		*Ref*	
***Individual dispositional factors***						
**Sex**						
Male	*Ref*	*Ref*	*Ref*	*Ref*	*Ref*	*Ref*
Female	3.67 (1.93,6.98)	3.63 (1.73, 7.61)***	1.60 (0.98, 2.62)	1.51 (0.95,2.40)	2.16 (1.21,3.88)	1.96 (1.04, 3.69)*
**Age group (years)**						
18–34	*Ref*	*Ref*	*Ref*	*Ref*	*Ref*	*Ref*
35–59	1.29 (0.55, 0.03)	1.00 (0.41, 2.47)	1.32 (0.68, 2.57)	1.24 (0.66, 2.32)	0.95 (0.47,1.95)	0.84 (0.41,1.68)
60 and above	1.67 (0.65, 4.28)	0.85 (0.29, 2.46)	1.41 (0.55, 3.57)	1.15 (0.50, 2.64)	1.00 (0.44, 2.28)	0.74 (0.31,1.72)
**Marital status**^**a,b,c**^						
Not married	*Ref*		*Ref*		*Ref*	
Married	1.09 (0.55,2.13)		0.83 (0.44, 1.56)		0.84 (0.46, 1.52)	
**Ethnicity**^**a**^						
Malay	*Ref*		*Ref*	*Ref*	*Ref*	*Ref*
Non-malay	0.96 (0.53,1.75)		2.16 (1.28, 3.67)	2.15 (1.29,3.58)**	1.72 (0.95, 3.13)	1.77 (0.99,3.16)
**Education level**^**a,b,c**^						
No formal education	1.98 (0.46, 8.53)		0.93 (0.33, 2.60)		1.16 (0.31, 4.29)	
Primary	1.35 (0.48,3.77)		1.54 (0.62, 3.82)		1.02 (0.41, 2.58)	
Secondary	1.17 (0.55,2.49)		1.45 (0.65, 3.24)		1.12 (0.53, 2.35)	
Tertiary	*Ref*		*Ref*		*Ref*	
**Employment status**^**b**^						
No	1.77 (0.99,3.19)	0.85 (0.41, 1.75)	1.28 (0.71, 2.32)		1.46 (0.84, 2.56)	1.12 (0.60,2.08)
Yes	*Ref*	*Ref*	*Ref*		*Ref*	*Ref*
**Household income group**^**b,c**^						
Low	1.62 (0.58,4.48)	2.11 (0.94, 4.74)	1.23 (0.46, 3.30)		1.57 (0.73, 3.40)	
Middle	0.88 (0.32,2.36)	2.15 (0.95, 4.89)	1.32 (0.51, 3.44)		1.15 (0.52,2.54)	
High	*Ref*	*Ref*	*Ref*		*Ref*	
**Residency**^**a**^						
Urban	0.99 (0.55,1.79)		1.46 (0.87, 2.45)	1.36 (0.81, 2.29)	1.58 (0.87,2.89)	1.48 (0.83,2.65)
Rural	*Ref*		*Ref*	*Ref*	*Ref*	*Ref*
**Received training**						
No	*Ref*	*Ref*	*Ref*	*Ref*	*Ref*	*Ref*
Yes	1.93 (1.05, 3.54)	2.10 (1.10,4.00)*	1.84 (0.95,3.54)	2.07 (1.10, 3.89)*	1.84 (1.02,3.33)	2.19 (1.22,3.93)**
**Duration of care**^**b**^						
Less than 2 years	*Ref*	*Ref*	*Ref*		*Ref*	*Ref*
2 years and more	1.87 (1.11, 3.16)	1.91 (1.08, 3.37)*	1.03 (0.57,1.88)		1.87 (1.05,3.32)	1.91 (0.98,3.70)
**Intensity of care (in a week)**^**a,b,c**^						
19 h and less	*Ref*		*Ref*		*Ref*	
20 h and above	0.78 (0.42, 1.44)		1.11 (0.61, 2.02)		1.19 (0.65,2.19)	
**Self-reported illness**^**b,c**^						
No	*Ref*	*Ref*	*Ref*		*Ref*	
Yes	1.64 (0.89, 3.03)	1.53 (0.84, 2.76)	1.18 (0.61, 2.30)		0.92 (0.52, 1.64)	
**Perceived health status**^**b,c**^						
Very poor to fair	1.86 (0.97, 3.59)	1.58 (0.77, 3.27)	0.97 (0.55, 1.73)		1.29 (0.67,2.47)	
Good to excellent	*Ref*	*Ref*	*Ref*		*Ref*	
**Presence of DM, HPT or HPC**^**b,c**^						
No	*Ref*	*Ref*	*Ref*		*Ref*	
Yes	2.03 (1.08, 3.8)	1.74 (0.82, 3.70)	1.12 (0.61, 2.06)		0.92 (0.50,1.69)	
***Contextual factors***						
**Living in the same household**^**a,b,c**^						
No	*Ref*		*Ref*		*Ref*	
Yes	1.09 (0.38, 3.12)		0.94 (0.43,2.02)		1.31 (0.46,3.72)	
**Had assistance**						
No	2.12 (1.14, 3.93)	1.74 (0.88, 3.45)	1.99 (1.01, 3.93)	2.02 (1.06,3.83)*	1.87 (1.01,3.45)	1.66 (0.92,3.00)
Yes	*Ref*	*Ref*	*Ref*	*Ref*	*Ref*	*Ref*

*CI* Confidence Interval, *OR* Odds Ratio, *Ref* Reference category, *DM* Diabetes Mellitus, *HPT* Hypertension, *HPC*, Hypercholesterolemia; ^a^ *p*-value > 0.25 for univariate analysis effect on health; ^b^ *p*-value > 0.25 for univariate analysis effect on work; ^c^ *p*-value > 0.25 for univariate analysis effect on social; * *p*-value < 0.05; ** *p*-value < 0.01; ***p-value < 0.001; ^#^ including other assistance. Area under ROC curve for model effect on health = 0.7495; Area under ROC curve for model effect on work = 0.6716; Area under ROC curve for model effect on social = 0.6876

The factors associated with the effects on work were ethnicity, received training and had no assistance to provide the care. Non-Malay caregivers (OR = 2.15, 95% CI [1.29, 3.58]) were more likely to have their work affected as compared to Malay caregivers. Caregivers who received training and had no assistance were 2.07 (95% CI: 1.10–3.89) and 2.02 (95% CI: 1.06–3.83) times more likely to have their work affected, as compared to caregivers who did not receive training and had assistance, respectively. Factors associated with the effects on social activities were sex and received training. Female caregivers and caregivers who received training were 1.96 (95% CI: 1.04–3.69) and 2.19 (95% CI: 1.22–3.93) times more likely to have their social activities affected, as compared to male caregivers and caregivers who did not receive training, respectively. The condition indexes of all variables were less than 30 (ranging from 17.04 to 24.15), implying that multicollinearity was unlikely. Since the AUC for each model was greater than 0.6, the models were considered fit.

## Discussions

Our study showed that 5.7% of the adult population in Malaysia were informal caregivers. Caregivers were mainly females, aged 36–59 years old and reported illness in the past 2 weeks. Care recipient’s need for personal care and individual dispositional factors of sex, received training, and duration of care were associated with an effect on health of caregivers. Individual dispositional factors of ethnicity and received training as well as contextual factor of having no assistance were associated with effect on work. Individual dispositional factors of sex and received training were associated with effect on caregiver’s social activities.

### Factors associated with informal caregiving

In our study, only individual dispositional factors of sex, age group, and self-reported illness were significantly associated with provision of informal care. The association of female sex and caregiving is consistent with other studies worldwide [39–41]. Family caregiving has its roots as a tradition and social value representing filial piety in Malaysia. The female members of the family are often brought up to manage household chores and caregiving responsibilities while upbringing of the males concentrated on providing financial support for the family [15, 42, 43]. Our finding of more respondents from the older age group providing informal care than the younger age group is a cause of concern as it reflects the reality that the elderly had to care for the elderly. Ability to provide care are affected as age increases because there would be a decline in strength and mobility in carrying tasks [32]. Studies have found a higher amount of care provision among caregivers as one ages [44, 45]. In Malaysia, young adults migrate to the cities for better job opportunities [46], leaving the older generation behind. This resulted in the shift of caring responsibility to the care recipient’s spouse or other senior family members [14]. This study also found that those who provided informal care were more likely to report presence of an illness. The task of providing care to the sick is very demanding and may cause deterioration in their health status, both physically and psychologically [47]. These results concurred with the findings from South Australia where an increased risk of chronic conditions was found among caregivers [48]. Therefore, strengthening the existing support groups and services in the healthcare system in Malaysia is crucial to help the population cope with their caregiving responsibilities.

### Effect on health

In our study, caregiver’s need for personal care as well as individual dispositional factor of sex, previous training experience, and duration of care were significantly associated with effect on health of informal caregivers in Malaysia. Females, those who had previous training, and provided care for 2 years and more were more likely to be affected in terms of health. Park et al. [49] indicated that caregivers may be susceptible to neglect their health due to time constraints to cater for caregiving responsibilities. Provision of personal care such as walking, feeding, dressing, toileting and bathing have been associated with longer hours of care which may lead to reduced caregiver’s time and motivation for self-care. This has resulted in increased physical and mental health-related stress among caregivers [50–53]. The sandwich generation refers to adults who provide simultaneous care to their young or adolescent children and an older family member or friend [54]. Women of the sandwich generation in Malaysia has adopted a new trend of responsibilities where they juggle between their formal job and caregiving responsibilities [55]. Caregivers require assistance, emotional support, and time off from their caregiving responsibility [56–58] but these needs are hard to meet especially when one has to juggle between formal job and caregiving responsibilities. As such, these women may experience burnout which affects their health physically and mentally [17, 24, 27–30, 57].

Provision of training to caregivers was proven beneficial for their caregiving tasks [59]. However, our study indicated that caregivers with previous training were more likely to be affected health-wise compared to those without previous training. A higher sense of obligation to attend to the demanding responsibilities of caregiving when one is trained to provide care could be a possible explanation to the greater amount of stress experienced, leading to negative consequences to their health [60]. In terms of duration of care, the average in the USA was 4 years [61] and those who cared for patients with dementia provided care for one to 4 years more compared to caregivers of patients with illness other than dementia [62]. Our study showed an association between longer duration of care and effect on health which is consistent with a study that indicated worse caregivers’ health with longer duration of care provision [63]. The need to provide long term care coupled by the possibility of reduced working hours, absenteeism, decreased job performance, or early retirement as a result of caregiving commitments may lead to physical and mental fatigue [64]. Abu Bakar et al. [17] have expressed the need to establish a strong support system to help informal caregivers cope with caregiving responsibilities in Malaysia. Support services in Malaysia mainly centered around home help services and home nursing services [65]. Countries in the West have implemented policies catered to improve caregivers’ physical and mental well-being by providing specific support services, including training/education, respite care and counselling [66, 67]. With the establishment of a systematic and strong informal care support system and network, duration and burden of care could be minimised.

### Effect on work

Our study found significant association between individual dispositional factors of ethnicity and previous training experience, as well as the contextual factor of having assistance in caregiving responsibilities with the effect on caregivers’ daily work. Caregiving has been shown to have an overall impact on the daily work of caregivers due to altered sleeping and eating habits [54]. Work interference or a change in work status among caregivers due to caregiving demands have been reported [61, 68]. Fitting caregiving responsibilities into work schedule may be a struggle for caregivers [69]. In order to adapt, many caregivers opt for part-time jobs which offer less income, security and career prospects than a full-time job [70] while others had to resign, opt for early retirement, or give up career opportunities to commit fully to their caregiving responsibilities [70]. For caregivers who managed to incorporate their caregiving responsibilities into their work pattern, a risk of poorer job performance still exists which may reduce their chance of a promotion. Choo et al. [71] found that Chinese caregivers experienced more burden from caregiving as compared to Indian and Malay caregivers in Malaysia. Malays in Malaysia who are mostly Muslims perceived caregiving as less stressful as compared to non-Malays, and hence had a lower level of burden compared to non-Malays [72]. According to the Islamic faith, Muslims were required to be content and satisfied with what was bestowed to them in life, even during times of hardship and uncertainty as one’s life and fate has been determined by Allah [73]. Therefore, they should accept Allah’s will and perform their caregiving responsibilities and the burden that comes with it with an open heart [17]. The higher sense of burden among Chinese caregivers, which are the second major ethnic group in Malaysia could have contributed to the higher likelihood in effect on other daily work of the non-Malay group [71]. Studies have shown a positive effect of training to caregivers [59, 74], but our study found that caregivers with previous training experience were more likely to be affected in their daily work compared to those without. This could be due to the heavier burden of care to those who are trained leading to problem on focusing and performing their work. Insufficient resources such as knowledge, skills, social support as well as respite and community services could elevate the challenges of caregiving [42]. Contextual factors such as inadequate work or family support have also been found to be strongly associated with exhaustion [75]. A cross-sectional study of family caregivers of patients with dementia found that informal support helps lower caregivers’ burden while formal support such as assistance from maids and private nurses did not reduce caregivers’ burden [71]. In our study, daily work of caregivers without assistance were more likely affected compared to caregivers with some form of assistance, confirming that inadequate support increases challenges faced by caregivers. Hence, workplace support and flexible employment policies for informal caregivers which does not affect seniority or rates of pay as well as support from family, social network, and community care services is crucial to reduce the negative consequences of informal caregiving in Malaysia [76].

### Effect on social activities

Ideally, a balance between the responsibilities and rewards from a caregiving role could have a positive effect on caregivers. However, increasing demands and expenditure for long-term care could be overwhelming and restricts caregivers from other aspects of their life [77]. Fatimah et al. [78] reported that caregivers had to cope with various mental, financial, physical, and social problems; the worst being social isolation. Osman et al. [79] added that caregivers are often affected emotionally, frustrated, and burnout due to caregiving responsibilities, which disrupts communication in the family. In this study, females and those with previous training experience were more likely to be affected negatively in social aspects. Female caregivers were more often obligated to care for the needs of the family rather than themselves [40, 80, 81] and often receive less social support than male caregivers [82–84]. As such, they may neglect social needs in their life and substitute their social time for caregiving responsibilities. Caregivers need opportunities for a break, practical help, someone to talk to about their own emotional needs and information about benefits and services to sustain the provision of caregiving [57, 58]. A support system from family and social network such as neighbour, friends, and family as well as community care services to temporarily take over the caregiving responsibilities of informal caregivers, women in particular, is necessary to facilitate time for the caregivers to participate in social activities which could be beneficial for their mental and physical well-being.

In contrast to caregivers who had not been trained, the well-being, career, and social activities of those who were trained were more likely to be affected in our study. This surprising finding contradicts those from other studies [59, 74]. One may hypothesize that training does not directly affect the health, work, or social activities of caregivers, although it may enhance the caregiver’s knowledge and skills in providing care. Training of caregivers have been shown to be useful in ensuring that the caregivers carried out their duties and responsibilities successfully [59]. In Malaysia, the provision of training for caregivers are specified in the policy and plan of action for the elderly [85, 86]. However, the training provided may not be sufficient or specific to address the needs of the caregiver [60]. The informational needs of caregivers and the negative effects of those needs not being fulfilled have been documented previously [87]. Provision of resources that is specific to the needs of the caregiver is crucial to provide leverage to their caregiving responsibilities. As such, health care professionals play a pivotal role in connecting informal caregivers to the services they need based on their distinct and varied needs. Nevertheless, this finding warrants further studies to assess the unmet training needs among the caregivers in Malaysia.

### Study limitations and future research

This study has many strengths. It is the first nationwide study on informal caregivers in Malaysia where a relatively large sample size and validated instruments were used. Hence, it contributes to the body of knowledge concerning the impact of caregiving in Malaysia since we currently have very little information on this aspect of care. However, several limitations exist. As this is a cross sectional study causality cannot be determined. There may be confounding factors such as severity of illness or condition of the care recipient and relationship between caregivers and care recipients which were not considered in this study and these issues could be topics to be focused upon in future research.

## Conclusion

This cross-sectional study describes the factors associated with informal caregivers and the effects of the caregiving roles on their health, work and social activities. Individual dispositional factors of sex, age, and self-reported illness were associated with provision of informal care in Malaysia. Informal caregivers faced effects on their health, work, and social activities which may be detrimental to their well-being. This understanding is crucial for planning of support for caregiver and future policy making.

## Data Availability

To ensure participants’ privacy, the datasets generated and analysed in this article is not made publicly available. Request for data can be obtained from the Head of Centre for Biostatistics & Data Repository, National Institutes of Health, Ministry of Health Malaysia on reasonable request and with the permission from the Director General of Health, Malaysia.

## Abbreviations

NHMS: National Health and Morbidity Survey
DM: Diabetes mellitus
HPT: Hypertension
USA: United States of America
OR: Odds Ratio
CI: Confidence Interval
NGOs: Non-Governmental Organizations
DOSM: Department of Statistics Malaysia
EBs: Enumeration Blocks
LQs: Living Quarters
MREC: Medical Research and Ethics Committee
NMRR: National Medical Research Register
NCD: Non-communicable disease
PSU: Primary Sampling Unit
SSU: Secondary Sampling Unit
